# Pain associated with breast cancer: etiologies and therapies

**DOI:** 10.3389/fpain.2023.1182488

**Published:** 2023-12-11

**Authors:** Lisa V. Doan, Jenny Yoon, Jeana Chun, Raven Perez, Jing Wang

**Affiliations:** ^1^ Department of Anesthesiology, Perioperative Care, and Pain Medicine, NYU Grossman School of Medicine, New York, NY, United States; ^2^ Department of Neuroscience and Physiology, NYU Grossman School of Medicine, New York, NY, United States

**Keywords:** breast cancer, cancer pain, pain management, post-surgical pain, neuropathy

## Abstract

Pain associated with breast cancer is a prevalent problem that negatively affects quality of life. Breast cancer pain is not limited to the disease course itself but is also induced by current therapeutic strategies. This, combined with the increasing number of patients living with breast cancer, make pain management for breast cancer patients an increasingly important area of research. This narrative review presents a summary of pain associated with breast cancer, including pain related to the cancer disease process itself and pain associated with current therapeutic modalities including radiation, chemotherapy, immunotherapy, and surgery. Current pain management techniques, their limitations, and novel analgesic strategies are also discussed.

## 1. Introduction

### 1.1. Breast cancer overview

Breast cancer is the most commonly diagnosed cancer, accounting for 29% of newly diagnosed cancers in the United States. It is the second leading cause of cancer death in women in the United States. According to the American Cancer Society, in the year 2023, over 297,000 new cases of female breast cancer and over 43,000 deaths are expected in the United States alone (1). Over the last few decades, the incidence of breast cancer increased and is expected to continue to grow. This is partially explained by improved screening and detection strategies as well as significant growth of an aging population. In the year 2020 alone worldwide, there were 2.3 million new diagnoses and 685,000 deaths (2–5). Current global projections predict that new diagnoses will reach 2.7 million annually with 870,000 deaths by the year 2030 (6).

### 1.2. Breast cancer pain overview

For patients with breast cancer, the burden of illness includes physical, emotional, and psychological distress. The prevalence of pain among patients with cancer is significant and depends on a variety of factors, including patient demographics, cancer type and extent, and treatment interventions. Pain is strongly associated with patients’ experience of breast cancer, as it is a common consequence related either to the disease pathology itself or to therapies, including both surgical and non-surgical interventions (Table 1) (20).

**Table 1:** Etiology of pain.

	Symptoms/syndromes	Incidence in % (reference)
Breast cancer specific factors		
Local cancer		
• Early stage	Does not typically involve pain	N/A
• Advanced stage	Dependent on size of tumor and involvement with chest muscles, and ribs	39–66 (7)
Metastatic cancer	Bone, brain, liver, lungs	66–86 (7)
Treatment related factors		
Chemotherapy		
• Platinum-based agents such as cisplatin and carboplatin	Chemotherapy-induced peripheral neuropathy	30–68 (8)
• Taxanes such as docetaxel and paclitaxel	Taxane acute pain syndrome	2.8–72 (9–12)
Immunotherapy		
• Monoclonal antibodies	Infusion related flu-like symptoms	<1.1–17 (13)
• Immune checkpoint inhibitors	Infusion related flu-like symptoms	<1.1–17 (13)
Radiation therapy		
	Radiation-induced brachial plexus neuropathy	24–47 (14)
	Radiation dermatitis	30–95 (15)
Hormonal therapy		
• Aromatase Inhibitors	Myalgia and arthralgia	5–28 (16)
• Tamoxifen	Myalgia and arthralgia	4–21 (16)
Breast Surgery		
• Lumpectomy, total mastectomy, lymph node dissection	Post-mastectomy pain syndrome (inclusive of neuropathic pain)	20–60 (17–19)
• Total mastectomy	Phantom breast pain after total mastectomy	13–44 (18)

## 2. Methods

Inclusion criteria for this narrative review includes literature published in the English language between 2000 and the present. Primary literature search was conducted in PubMed and Google Scholar. The following search terms were used:

(“Pain”) and “breast cancer” or “risk factors” or (“chemotherapy” and “breast cancer”) or (“immunotherapy” and “breast cancer”) or (“radiation” and “breast cancer”) or (“Metastases” and “breast cancer”) or (“surgery” and “breast cancer”)

## 3. Predictors for development of breast cancer pain

Cancer-related pain tends to be associated with a complex interaction of factors including but not limited to disease pathology, genetics, lifestyle influences, and psychosocial stressors. For patients with breast cancer specifically, pain can arise in a variety of ways including symptomatic pain and pain related to cancer therapy. Understanding and identifying risk factors for pain will not only help prevent painful experiences for patients but also guide treatment strategies.

### 3.1. Patient-related predictors

Patient-specific risk factors for breast cancer pain include not only socio-demographics such as age and education level, but also medical, psychological, and behavioral factors.

### 3.2. Socio-demographics

Young age is one socio-demographic risk factor associated with breast cancer pain. The correlation between young age and breast cancer pain may be partially due to more advanced local disease at time of diagnosis (21). Other potential factors linking young age and breast cancer pain include changes in pain perception, subjective experiences of pain, differing levels of physical activity, and more invasive therapeutic interventions in younger patients (21). The literature supporting the predictive value of age in breast cancer pain primarily refers to pain related to both surgical and non-surgical treatment, rather than primary pain (17, 22–28).

Low socioeconomic status and low education level are also risk factors for breast cancer pain. Breast cancer patients of lower socioeconomic status in terms of income and net wealth are more likely to report greater chronic pain post-treatment (23, 25, 29). While the predictive value of low socioeconomic status could not be demonstrated over time, studies have shown that breast cancer survivors with lower education levels, defined as less than or including 13 years, are more likely to report chronic pain even 7–9 years post-treatment (25, 30–33).

A meta-analysis of risk factors of pain in breast cancer survivors demonstrated no association in chronic pain development in breast cancer patients with or without children, or patients that were in a relationship or single (30–32).

### 3.3. Medical comorbidities

As with many cancers, breast cancer on average affects an older population. Due to their age, these patients may experience multiple co-morbidities, including prior or existing pain syndromes. Patients with previous pain comorbidities significantly reported more pain associated with breast cancer treatment compared to patients with no illnesses that previously caused pain (17, 30). These comorbidities included back pain, arthritis or arthrosis, fibromyalgia, and neck pain in addition to frozen shoulder, sciatica, migraine, and systemic lupus erythematous (17, 30).

Diabetes has been linked to breast cancer symptoms as well. One study found that breast cancer survivors with a comorbid diabetes diagnosis had poorer physical function and greater fatigue than their counterparts without diabetes (34). Another found that breast cancer survivors with diabetes were more likely to experience tenderness at surgical sites (35).

### 3.4. Genetics

Genetics may also play an important role in pain perception for cancer patients. Several genes are involved with the inflammatory pathway that contributes to tumor growth and spread, while the cancer itself can also have perineural involvement and release chemokines and cytokines that result in increased sensitization of peripheral nerve terminals and possibly central neurons as well (36, 37). Specifically, algogenic mediators and pro-inflammatory cytokines including interleukin (IL)-1 and tumor necrosis factor (TNF)-α are known to induce inflammatory pain (38).

Studies have demonstrated associations between variations in cytokine genes and the development of pain in patients with breast cancer. For example, one study demonstrated breast cancer patients who were carriers of a minor allele for IL-receptor-1 (ILR1) had a 53% decrease in odds of reporting pre-operative breast pain (37). This is consistent in mice, where removal of IL1R1 function led to a decrease in inflammation and pain behavior (39). Regarding post-operative breast pain, a particular study outlined three small nuclear polymorphisms (SNPs), IL-6, CXCL-8, and TNF, to be associated with differing breast pain phenotypes after surgery. For example, a rare G allele of IL-6 was associated with decreased serum IL-6 concentrations, preventing the development of mild persistent breast pain after oncologic surgery while a common T allele of CXCL-8 was associated with increased serum CXCL-8 and promotion of mild persistent breast pain after oncologic surgery (38).

### 3.5. Psychological conditions

The relationship between pain and psychological comorbidity such as depression and anxiety that influence pain perception after breast cancer treatment is well known (17). Patient-related psychiatric and psychological risk predictors such as post-traumatic stress disorder, low mood, and anxiety commonly co-exist and influence development of breast cancer-related pain (40). Pain and depression has been established to have a reciprocal relationship as one complicates the nature and management of the other (41). Additionally, psychological distress, measured by standardized depression and anxiety inventories, was found to be predictive of moderate-to-severe acute and chronic post-surgical breast cancer pain, while psychological robustness regarding emotional and cognitive resilience was found to be protective, albeit temporarily, for recovery trajectory (27, 42). Furthermore, there are several studies focusing on individual distress predictors such as fear and catastrophizing that are strongly correlated with chronic pain (43, 44). Pain-related fear also plays role in predicting pain (45).

### 3.6. Behavioral factors

Behavioral predictors include lifestyle habits such as obesity, substance use, and physical activity. Studies revealed that people with body mass index (BMI) greater than 30 kg/m^2^ had 1.33 times higher odds for developing chronic pain related to breast cancer treatment, compared to those with BMI less than 30 (95% CI 1.08–1.67, *p* = 0.008) (17, 25, 31, 32, 46, 47). This may be explained by obesity being associated with elevated pro-inflammatory cytokines that may correlate with chronic pain and inflammatory states (21). Regarding substance use, alcohol use is associated with a significantly lower chance of chronic pain development (31, 32). Multiple studies reveal that smoking cigarettes is associated with increased persistent breast cancer pain (28, 31). Predictors for lower pain frequency include moderate exercise and non-sedentary activity (28, 46, 48–50). Specifically for breast cancer patients, combined training, which includes aerobic and resistance exercises, successfully reduced pain intensity and improved quality of life (51).

## 4. Breast cancer-specific factors

### 4.1. Local vs. metastatic cancer

Localized breast cancer in its early stage does not typically involve pain (Table 1) (52, 53). Pain increases as breast cancer advances (53). Pain can become severe in localized breast cancer depending on the size of the tumor and its involvement with the chest, more specifically the muscles and ribs (53). The larger the tumor, the more physical compression that is exerted on the tissue in its immediate surroundings (53). This can ultimately lead to tissue injury and increased inflammation (52, 53).

While breast cancer is localized to the breast at presentation in 61% of cases, it becomes regionally advanced in 32% and metastatic in 7% of cases (54). Bone is the first metastatic site for up to 40% of breast cancer patients (55–58). Bone metastases are also most associated with inflicting pain in women with metastatic breast cancer (59, 60). Both osteolytic and osteoblastic lesions can result in pain (59). The highly vascularized bone marrow is a source of growth factors and blood vessels, fostering an optimum environment for tumor cell nourishment and growth (59). Breast cancer cells produce molecules similar in structure to parathyroid hormone (PTH), which promote cells that build up or break down bone (59). These parathyroid hormone-related peptides have a strong affinity to bone marrow cells (59, 60). This interaction triggers a destructive symbiotic relationship as an increase in the growth of tumor cells directly aids in the formation of osteoclasts responsible for the dissolution and absorption of bone (59, 60). Osteoclastogenesis triggers more bone resorption, releasing a significant amount of growth factors from the bone matrix that further activate the tumor cells and cyclically exacerbate bone destruction (59). This cycle of bone loss can lead to painful pathological fractures, hypercalcemia and nerve compression (52, 59–61).

In addition to osteolytic features, bone metastases in breast cancer also feature osteoblasts, which synthesize and form bone tissue (59, 61). The cycle of tumor cells causing osteoclasts to degrade bone tissue also results in upregulating osteoblasts to lay down new bone tissue (59, 61). Unfortunately, with advanced metastatic bone cancer, these osteoblasts tend to replace the bone matrix and rebuild bone in random locations (59–61). This random build-up disrupts the balance of the bone microenvironment, as these two osteocytes can work alongside each other on the same bone, with a portion of the bone mostly degraded by osteoclasts and another portion dangerously built up with excessive bone matrix and minerals by the osteoblasts (60, 61). There is less research done regarding the role of osteoblasts in breast cancer metastases, as compared to the role of osteoclasts, which are the main cause of pain related destruction in the bone (60, 61). Both osteolytic and osteoblastic lesions are associated with nociceptive, localized pain that are characterized as deep, sharp, and non-radiating (59, 60).

Two-thirds of patients diagnosed with metastatic cancer have reported symptomatic pain, including neuropathic pain, nociceptive pain, or a combination of the two (62). The humerus is one of the most common sites for breast cancer to spread (56). The pain experienced in the humerus is mainly due to resulting pathological fractures in the absence of trauma (56). Breast cancer metastases also favor trabecular bone due to its large surface area that exposes it to bone marrow and blood flow, making it an ideal microenvironment for tumor-cell invasion and survival (61). Bone metastases are usually located in irregularly shaped bones (61). Other bones where cancer commonly metastasizes to consist of the spine, pelvis, femur, ribs, and skull (61). Tumor cells in the spine are particularly dangerous as they can compress the spinal cord and cause nerve damage and in extreme cases, paralysis (61).

In addition to bones, solid tumors resulting from breast cancer tend to metastasize to the brain, liver, and lungs (53, 61). In rare cases, breast cancer can even metastasize to the urinary tract, peritoneum, or bladder (53, 63). The invasion of malignant tumors in these vital organs can cause mass effect leading to nociceptive pain, as well as painful localized inflammation, tissue and nerve damage, the latter resulting in neuropathic pain (53). For example, metastases to the brain can result in painful headaches (53).

Since advanced breast cancer patients live in a constant state of inflammation, cytokines also play an important role in the experience and mediation of pain (52). Studies that have focused on the connection between breast cancer pain and the amount of cytokines produced in patients have noted that women with more advanced breast cancer have significantly higher quantities of cytokine production, and report higher severities of pain associated with their cancer, as opposed to women with localized breast cancer (52).

## 5. Treatment-related factors

### 5.1. Chemotherapy

Chemotherapy results in both survival benefits and reductions in mortality in breast cancer patients (64). However, chemotherapy-induced pain, specifically chemotherapy-induced peripheral neuropathy (CIPN) is a clinically significant side effect of chemotherapy (9). The odds for development of chronic pain in patients treated with chemotherapy are 1.44 times compared to those who did not undergo chemotherapy (95% CI 1.23–1.69, *p* < 0.00001) (28, 30, 31, 33, 65, 66). Another study found that the prevalence of persistent pain was higher in women who received chemotherapy after surgery than women who did not (14.5% vs. 8.4%, *p* < 0.01) (65).

Breast cancer chemotherapies most commonly associated with dose-dependent CIPN include platin compounds like cisplatin and carboplatin, as well as taxanes like docetaxel and paclitaxel (67–69). CIPN is often sensory in the early process with patients reporting tinging and numbness of the feet or fingers (67–69). Additional sensory symptoms include ataxia and gait disorders of the lower extremities, as well as painful sensations including paresthesia, dysesthesia, tingling, itching, burning, tight, stabbing, and aching (67). Several factors that predispose to peripheral neuropathy due to chemotherapy include diabetes mellitus, alcohol use, inherited neuropathy, preexisting neuropathy, age-related axonal loss, and prior chemotherapy (67, 70). Generally, non-severe taxane-induced peripheral neuropathy improves significantly after discontinuation of the treatment (71). Unfortunately, a prospective trial of patients with high-risk breast cancer found that 44.8% of patients treated with docetaxel met diagnostic criteria for CIPN one year after treatment (8). Another meta-analysis revealed CIPN prevalence at 68.1% within the first month of treatment, 60.0% at three months, and 30.0% at six months of treatment (8).

Taxane acute pain syndrome (TAPS) is chemotherapy-induced pain due to taxane chemotherapy with an unreliable incidence between 2.8% and 72% in breast cancer patients, perhaps due to the underestimation and inconsistent definition of the phenomenon (10–12). Despite this, TAPS is described as diffuse, nonlocalized arthralgia and myalgia, and multiple studies have found that it is at its maximum at around three days of treatment with decline of pain after around five to seven days (9–12). TAPS incidence may also depend on the dosing and frequency of chemotherapy. One study found that 26% of patients with docetaxel-induced myalgia and arthralgia required a dose reduction from the initial dose of 100 mg/m^2^ (72). Other studies have found that TAPS incidence was also higher in patients receiving three weekly treatments of paclitaxel compared to patients receiving one weekly treatment (73–75).

In addition to direct neurotoxic damage, chemotherapy can also result in the adverse effect of impaired muscle function, weakness, and wasting (76, 77). Breast cancer patients undergoing chemotherapy specifically were found to have worse muscle function compared to healthy noncancer peers (76, 77). A systematic study revealed that early-stage breast cancer patients lost 1.3 kg lean body mass and continued to lose body mass during and after adjuvant chemotherapy (78). Compounded with worse muscle function, breast cancer patients may continue to experience joint and muscle pain that significantly affects their quality of life even years after completion of chemotherapy (69).

### 5.2. Immunotherapy

While breast cancer was once thought to be immunologically quiescent, recent findings supporting the immunogenicity of breast cancer have led to expansion of the use and study of immunotherapy as a viable breast cancer treatment (79). To date, the most established form of immunotherapy is the application of monoclonal antibodies. Monoclonal antibodies can treat breast cancer through direct elimination of tumor cells, activation of the immune cells to target tumor cells, or vascular disruption (80). Despite the demonstrated efficacy of monoclonal antibody treatment in reducing breast cancer recurrence and mortality, however, this therapeutic avenue may result in infusion- and inflammation-associated pain. Trastuzumab, a HER2 targeting recombinant monoclonal antibody, is among the first of targeted therapies for breast cancer and is now considered standard care for HER2-positive breast cancer patients (81). While generally well-tolerated, multiple clinical trials have found that patients may experience flu-like symptoms during and immediately after Trastuzumab infusion (82, 83). These symptoms, which can include headache, fever, shortness of breath, or nausea, are described as mild-to-moderate in severity, beginning during infusion and declining in severity in the days following infusion (84).

Immune checkpoint inhibitors (ICI) represent a rapidly developing subset of monoclonal antibodies. The research surrounding checkpoint inhibitors is promising, with one phase III trial demonstrating that atezolizumab, a PD-L1 targeting checkpoint inhibitor, increased both progress free survival and overall survival outcomes in untreated triple negative breast cancer patients (85). In addition to the infusion-related flu-like symptoms, clinical trials investigating checkpoint inhibitors for breast cancer have determined that this treatment is associated with arthralgia, fatigue, and skin toxicities, which may result in pain for patients (86). In regard to severity, most ICI-related adverse events are manageable with steroids and immunosuppressants, largely mitigating the need for dose-reduction (87). However, it is important to note that immunotherapy such as ICI inhibitors are often used in conjunction with chemotherapy for best results (80). This should be considered when assessing immunotherapy-associated pain.

Other potential avenues of breast cancer immunotherapies include therapeutic vaccines and oncolytic viruses. Because of their novelty, the research remains investigational and there is a dearth of research focusing on pain, especially in the long-term, that is associated with these therapies. In general, however, dendritic cell vaccines have been shown to be well-tolerated by patients (88, 89).

Despite these promising findings, one area of concern regarding immunotherapy is the possibility of psychological and neurological side effects, which can increase pain perception. For example, it has been shown that up to 10% of patients taking Trastuzumab develop depression (90). Furthermore, although not common, autoimmune encephalitis has been noted by multiple studies as an ICI-associated adverse effect (91, 92). Because both depression and neuroinflammation have been shown to exacerbate perceptions of pain, these adverse effects of immunotherapy should be further studied (93, 94).

### 5.3. Radiotherapy

Adjuvant radiation therapy improves survival rates and decreases risk of recurrence in patients with breast cancer. Despite this, radiation therapy is associated with acute and chronic side effects including fatigue, edema, skin fibrosis, and pain (95, 96). These symptoms depend on the irradiated volume and intensity of treatment but may have considerable impact on the potential development of neurotoxicity and neuropathic pain (95, 96).

Multiple studies demonstrate that adjuvant radiotherapy significantly increased the risk of patients reporting pain up to 1.5 times (24, 97). According to a recent study, 24%–47% of patients with breast cancer reported persistent pain of the irradiated area after radiotherapy completion (14). A prospective study demonstrated that pain related to breast radiotherapy peaked at 1-week following radiotherapy treatment regardless of dose or extent of the irradiated region. Additionally, patients younger than 59 years of age experienced more acute breast pain following radiation than patients over 60 years of age (14). The combination of both chemotherapy and radiotherapy is significantly associated with higher risk of breast cancer survivors reporting pain (30).

Radiation-induced brachial plexus neuropathy (RIBPN) is a peripheral neuropathic condition that can occur in breast cancer patients treated with radiation therapy to the chest wall, neck, or axilla, and can occur at variable times between six months to twenty years after radiation (98). While symptoms commonly initially present with paresthesia and pain, often the pain persists simultaneously as motor weakness and eventually, upper limb paralysis can occur (98). Another common source of pain after radiotherapy treatment is radiation dermatitis, a skin reaction of the irradiated area that occurs in up to 95% of patients (15). It consistently peaks around two weeks after radiotherapy, and can manifest in a variety of symptoms including erythema, edema, desquamation, and pain (99). A prospective study demonstrated that both overall pain and breast pain peaked after one week of radiotherapy, regardless of the radiotherapy dosage, fractionation, or extent of irradiated region. This may be attributed to the development of acute radiation dermatitis of the breast or chest wall closely following radiotherapy (14).

### 5.4. Endocrine/hormonal therapy

While neoadjuvant chemotherapy and radiotherapy has proven efficacious and become a standard of care in breast cancer treatment, endocrine therapy is also gaining traction in cancer therapies, demonstrating efficacy, high tolerability, and good compliance (100). Hormonal agents like tamoxifen and aromatase inhibitors do not seem to cause neuropathy (101, 102). However, multiple studies demonstrate that the use of hormone therapy is an independent predictor to long-term pain. These studies have postulated that musculoskeletal adverse effects related to the use of aromatase inhibitors or the increased incidence of breast fibrosis with the use of tamoxifen may be responsible for development of pain (69, 103–105). While the exact mechanism of hormone therapy-induced arthralgia is unknown, the sudden decrease in estrogen due to aromatase inhibition may be a possible hypothesis, as similar arthralgia is prevalent in peri- and post-menopausal women (106, 107). For aromatase inhibitor use, risk factors for the development of arthralgia include younger age, adjuvant chemotherapy, use of granulocyte colony-stimulating factor, and prior history of arthralgia, arthritis, or fibromyalgia. On the other hand, studies have demonstrated that at least half of patients experienced resolution of arthralgia around 6-months from onset of aromatase inhibitor therapy (106, 108). For tamoxifen use, patient are less likely to experience joint symptoms compared to aromatase inhibitor use. A study demonstrated that 75% of patients experiencing joint symptoms from aromatase inhibitor use transitioned to tamoxifen and obtained relief in symptoms (109).

Duloxetine has been studied in regards to reducing the pain associated with aromatase inhibitor use. Duloxetine is a serotonin noradrenalin reuptake inhibitor, and it enhances signaling through both serotonin and noradrenergic systems (110). It has been used widely for a range of neuropathic pain conditions including fibromyalgia, diabetic neuropathy, and trigeminal neuralgia (110–112). One study demonstrated that duloxetine relieved joint and musculoskeletal pain in breast cancer patients undergoing aromatase inhibitor therapy (113). A 2022 meta-analysis, however, found that the efficacy of duloxetine was similar to that of placebo (114).

In addition, some studies illustrate that endocrine therapies demonstrated an increased risk for more frequent pain, specifically in post-menopausal women who underwent surgical treatment (31).

### 5.5. Breast surgery

Surgical intervention for breast cancer treatment includes resection of the entire breast in a simple or radical mastectomy, or a partial mastectomy with or without surgical management of the axillary lymph nodes. Currently, advances in surgical treatment and efforts to reduce risk of recurrence strongly guides the increased use of mastectomy treatment (115, 116). Up to 60% of breast cancer survivors report persistent postsurgical pain, inevitably leading to reduced quality of life, impaired functionality, and need for therapeutic interventions (17–19). Post-surgical pain can be organized into acute and chronic pain with varying risk factors and symptoms.

Acute post-operative pain describes pain after surgical intervention within two weeks. The most consistent surgical risk factors associated with acute post-operative pain include axillary dissection and reconstruction, which are associated with 3–4-fold increased risk of moderate-severe pain and opioid use two weeks post-surgery (117–120). Additionally, longer duration of surgery and higher pain catastrophizing scores were found to be associated with increased severity of acute pain (121). The incidence of acute post-operative pain after breast cancer surgery is estimated to be between 61% and 67% (122). The underlying mechanism of acute post-mastectomy pain is likely caused by direct damage to tissue complicated by short-term, self-limited inflammatory changes at the surgical site (123). There may also be a component of tolerance or hyperalgesia in patients who are treated with short-acting opioid medications in the perioperative period (124). As such, there is a need to explore non-opioid management for acute post-operative pain because without treatment, there is high likelihood to progress to chronic post-operative pain.

Chronic pain induced by breast cancer surgery is termed post-mastectomy pain syndrome (PMPS), and it can involve phantom breast pain, intercostobrachial neuralgia, neuroma pain, or pain from nerve injury (53). In particular, mastectomies have a strong association with PMPS which is defined as pain that lasts at least three months after surgical intervention (125). Currently, the standard perioperative multimodal analgesia is modestly effective in PMPS prevention (126).

Numerous studies have explored the association with breast cancer surgery and chronic post-surgical pain (CPSP). The prevalence of CPSP in general is approximately 10% after all surgeries, with high intensity acute post-surgical pain being the strongest predictor for developing CPSP (127, 128). Regarding patients being treated for breast cancer, a study evaluating persistent pain following surgery, radiotherapy, and chemotherapy found that the greatest pain prevalence rate was in the post-surgery group (129). A large nationwide study demonstrated 58% of women experienced sensory disturbances in the surgical region even 1–3 years after surgery (24). Furthermore, a systematic review of observational studies reported an estimated median prevalence of persistent pain after breast cancer surgery at 37.5% at a median follow-up of 24 months (IQR 30%–51%) (97). A separate survey determined that 53% of breast cancer survivors suffered moderate-to-severe chronic pain after surgery and 18% suffered from severe chronic pain two years after surgery (24). The development of persistent pain after surgery involves a variety of mechanisms including direct tissue injury during surgery and inflammatory processes from surrounding tissue trauma. Postoperative healing can also result in inflammation, neuromas, and increased sensitivity due to nerves becoming entrapped in healing incisions (18, 130, 131). Meta-analyses demonstrated that patients with lymphedema after surgery were at 2.58 times higher odds of developing chronic pain than patients without lymphedema (28, 30, 33, 66).

### 5.6. Axillary surgery

A strong predictor of chronic pain after breast cancer surgery is axillary lymph node dissection (17, 97). The complex structure of the axillary region contributes to higher prevalence of chronic pain in patients with surgery in this area (132). A study demonstrated that axillary lymph node dissection was associated with a statistically significant increase of 1.77 times likelihood of pain compared with sentinel lymph node dissection, as well as significantly increased 4.97 times likelihood for sensory disturbances (24). Notably, the lateral cutaneous branch of the second intercostal nerve crosses the axilla and innervates the medial arm, and is the most commonly injured nerve during mastectomy surgeries, especially with axillary lymph node dissection (133). Axillary lymph node dissection was associated with 21% increase in the absolute increase in risk of chronic pain. Despite the risk of CPSP, the risks of omitting axillary surgery are also significant and include undertreating and reducing survival in breast cancer patients, as these dissections are confirmed methods to remove cancerous tissue and stage breast cancer accurately (97). Additional co-morbidities from axillary surgeries besides pain include reduced shoulder range of motion, upper extremity and hand weakness, lymphedema, and numbness (17, 134).

Compared with axillary lymph node dissections, sentinel lymph node biopsies are associated with fewer upper limb morbidities and no significant difference in survival (134, 135). Among the complications among breast cancer patients undergoing sentinel lymph node biopsies, sensory disorders were found to be the most common followed by pain (135). Patients with breast cancer undergoing sentinel lymph node biopsies are reported to have statistically significantly less pain, numbness, and shoulder movement restrictions at six months after surgery (136).

## 6. Breast cancer pain management

The World Health Organization (WHO) proposed an analgesic ladder in 1986 to guide pain treatment for cancer patients (137). While this guideline has undergone modifications throughout the years, it provides a simple structure for managing pain, reducing morbidity caused by pain in 70%–80% of patients (138). According to this analgesic ladder, cancer pain treatment should follow a sequential order from non-opioid drugs to weak opioids for mild-to-moderate pain to strong opioids for moderate-to-severe pain (137). Additionally, adjuvant medications such as tricyclic antidepressants, anti-convulsants, and corticosteroids may be utilized for different types of pain (53, 139). However, limitations to pharmacotherapy remain, as patients may develop unwanted side effects and even a ceiling effect often due to dose-limiting side-effects after chronic use (137).

As previously discussed, pain is usually not a symptom of breast cancer unless the cancer develops into its later stages. More often, patients report pain post-intervention. In this section, we will discuss pain management guidelines for specific causes of breast cancer pain (Table 2).

**Table 2:** Treatment of breast cancer pain.

	Therapy	Efficacy (references)
Breast cancer specific		
• Bone metastases	Radiation	53–86% of patients reported overall bone pain relief after radiation treatment (140)
	Radiation + bisphosphonate	Provided pain relief in 90%–100% of patients receiving this combination of treatment (140)
	NSAIDs (nonsteroidal anti-inflammatory drug)	Moderate/severe pain was reduced to mild pain after 1 week or 2 weeks with NSAID use in 26%–51% of patients (141)
	Opioids	Provides adequate pain relief in 70%–90% of the patients (141)
	Massage therapy	Provided pain relief in 35–38% of patients with bone metastases (142)
Treatment-related pain		
Chemotherapy induced peripheral neuropathy (CIPN)	Exercise	32.6–67.4% of patients reported exercise interventions significantly improved CIPN (143)
	Therapeutic massage	71% patients reported lower levels of acute chemotherapy related pain after treatment compared to baseline (144)
Hormonal therapy induced myalgia and arthralgia	Duloxetine	52%–64% of patients suffering from aromatase inhibitor associated musculoskeletal symptoms reported clinically meaningful (≥2 points) improvement of pain (113)
Acute postsurgical pain	Dexamethasone	40% reduction in the incidence of acute postop pain within 24 h (hours) (145, 146)
	Opioids	Provides adequate acute pain relief in 70%–90% of the patients (141)
	Intraoperative ketamine	Reduces postoperative pain scores 24 h after surgery in comparison to opioids alone (147)
	Paravertebral nerve block (PVB)	Preincisional PVB reduced the prevalence of acute and chronic postop pain over 12 months by 34–56% (148, 149)
	Pregabalin	Reduces acute postop pain in the recovery room (*p* = 0.01) among 4 clinical trials (150)
	Gabapentin	Reduces acute postop pain in recovery room (*p* = 0.04), and at 24 h (*p* = 0.72) among 8 clinical trials (150)
	Acupuncture	Relieves acute surgery-induced pain among 5 clinical trials (151)

CIPN, chemotherapy induced peripheral neuropathy; hr, hours; NSAID, nonsteroidal anti-inflammatory drug; PVB, paravertebral nerve block.

### 6.1. Metastatic pain management

As advanced breast cancer often involve metastasis to the bone, it is important to consider treatment of bone pain and skeletal-related events including fractures and future orthopedic intervention (152). Often, management includes analgesics medications including both opioid and non-opioid options, bone-targeted medications including osteoclast inhibitors, and adjuvants including corticosteroids and anti-convulsants (153). For patients with painful bony metastases that is difficult to manage pharmacologically, WHO guidelines recommend palliative radiation treatment, including both single and fractionated radiotherapy (140, 154). Massage therapy has also shown to be effective in relieving pain for patients with metastatic bone pain (142). Ultimately, however, opioids remain the first-line standard of care for palliative management of breast cancer pain (155).

### 6.2. Chemotherapy or radiotherapy pain management

Chemotherapy that prolongs survival in breast cancer patients often induce neurotoxic side effects including pain which is believed to be primarily affecting peripheral sensory nerves. Administration of fingolimod, a FTY720 pro-drug, has been demonstrated to enhance the chemotherapy benefits when treating triple negative breast cancer while also suppressing CIPN (156). In a double-blind, placebo-controlled trial of 206 breast cancer patients, those who received ganglioside-monosialic acid (GM1) also experienced less chemotherapy-induced peripheral neuropathic pain than counterparts receiving a placebo treatment (26.4% vs. 97.8%, *P* < 0.001) (157). Furthermore, neuropathic agents such as gabapentin and pregabalin have been studied in clinical trials as potential avenues for CIPN treatment. Gabapentin and pregabalin inhibit the alpha(2)delta-1subunit of voltage-gated calcium channels presynaptically at the first synapse of the primary sensory neurons in thedorsal horn of the spinal cord, which are known to be upregulated in nerve injuries (158). Clinically, studies investigating the efficacy of gabapentanoids seem to have mixed results, with some studies suggesting that gabapentanoids may decrease chemotherapy-induced myalgias in breast cancer patients, but more recent studies fail to support these findings (159–161).

In regards to non-pharmacological agents, therapeutic massage has been shown to reduce pain ratings and NSAID use as well as improve relaxation, mood disturbances, and fatigue in patients receiving cancer chemotherapy (144). Meta-analyses have demonstrated that acupuncture is not effective for chemotherapy or radiation-induced pain (151). Finally, a 2021 meta-analysis found that exercise improves CIPN symptoms (143).

### 6.3. Surgical pain management

Several options are commonly utilized in practice for management of surgical pain, including pharmacologic and interventional regional techniques (Table 3).

**Table 3:** Recent trials of therapies for breast cancer pain.

References	Condition	Study design	Sample size (*n*)	Treatment regimen	Comparator	Pain outcome	Findings
Fuster et al. (55)	Metastatic bone pain	Prospective open-label pilot study	40	Strontium-89 chloride (^89^Sr) injected intravenously (IV), with option to receive up to 3 doses	N/A	Responses 3 months (months) post-dose, graded as good (increase in functional status and decrease in pain score or analgesic score); partial (increase in functional status and decrease in pain score and no change in analgesics); no response	Response was good in 60% and partial in 32%
Briot et al. (109)	Anastrozole-induced musculoskeletal pain	Prospective, open-label, multicenter study	179	One mo after stopping anastrozole, letrozole 2.5 mg daily for 6 months	N/A	Percentage of patients who discontinued letrozole due to severe musculoskeletal pain (primary outcome)	At 6 mo, 71.5% [95% CI (confidence interval) 64.9, 78.1%] of patients were still taking letrozole. Overall, fewer women reported musculoskeletal symptoms after the switch
Jane et al. (142)	Metastatic bone pain	RCT (randomized controlled trial)	Massage therapy (MT) (48); Social attention (SA) (48)	45 min full body MT for 3 sessions	SA (presence of a caring therapist for 45 min) for 3 session	VAS (visual analogue scale) 20 min after each session (primary outcome)	Statistically significant improvement in VAS over time for MT vs. SA
Su et al. (157)	Taxane-induced peripheral neuropathy	RCT	Ganglioside-monosialic acid (GM1) (108); Placebo (108)	GM1 80 mg IV, day −1 to day 2 of taxane-containing chemotherapy (CT)	Placebo, day −1 to day 2 of taxane-containing CT	Functional Assessment of Cancer Treatment Neurotoxicity (FACT-Ntx) subscale at 2 weeks after completion of four cycles of taxane-based CT (primary outcome). Eastern Cooperative Oncology Group Neuropathy Scale (ENS; secondary outcome)	GM1 group scores were significantly better than placebo group scores. GM1 demonstrated a statistically significantly lower incidence of grade 1 or higher neurotoxicity when evaluating ENS sensory neuropathy (26.4% vs. 97.8%, *P* < 0.001)
Shinde et al. (161)	Paclitaxel-induced acute pain syndrome (P-APS)	RCT	Pregabalin (35); Placebo (35)	Pregabalin 75 mg twice daily through the 12 weeks of CT	Placebo twice daily through the 12 weeks of CT	Worst pain score for the week following 1st cycle of paclitaxel (primary outcome)	Worst pain scores were not significantly different between arms
Post-White et al. (144)	Chemotherapy induced pain	Randomized, prospective, 2-period, crossover intervention study	MT (11); Healing touch (HT) (67); Caring presence (P) (57)	4 weekly 45-min sessions of their assigned intervention (MT, HT, or P)	4 weekly sessions of a standard care	BPI (brief pain inventory) pain scores over 4 weeks (primary outcome)	Pain was lower in the MT (*P *< 0.001) and HT (*P *< 0.011) vs. P and control
Cortes-Flores et al. (145)	Acute postoperative pain	RCT	Dexamethasone (52); Placebo (52)	Dexamethasone 8 mg IV preoperatively	Placebo preoperatively	VAS 24 h (hours) postoperatively (secondary outcome)	Pain intensity was lower in the treatment group for all periods
Gomez-Hernandez et al. (146)	Acute postoperative pain	RCT	Dexamethasone (47); placebo (47)	Dexamethasone 8 mg IV preoperatively	Placebo preoperatively	VAS up to12 h postoperatively (primary outcome)	Dexamethasone treatment significantly reduced postoperative pain after surgery
Kairaluoma et al. (148)	Chronic postoperative pain	RCT	Paravertebral block (PVB) (42); Sham (42)	Preincisional PVB 0.5% bupivacaine 1.5 mg/kg	Sham with saline	VAS at 1, 6, and 12 mo (primary outcome)	Pain was lower in PVB group across all period
Gupta et al. (162)	Acute postoperative pain	RCT	PVB (37); Serratus plane block (SPB) (37)	PVB 20 ml of 0.5% bupivacaine	SPB 20 ml of 0.5% bupivacaine	VAS at 4, 6, 24, 48 and 72 h (secondary outcome)	Postoperative mean VAS scores were similar in both groups
Syal et al. (163)	Acute postoperative pain	RCT	Local anesthetic (LA) to incision site (34); PVB (34); pec blocks I & II (PEC) (33)	21 ml 0.5% bupivacaine with adrenaline wound infiltration at incision site (LA)	21 ml 0.5% bupivacaine with adrenaline PVB or PEC	VAS at 0, 2, 4, 6, 12 and 24 h (primary outcome)	VAS were lower in PVB group compared with others at 0, 2, 4, 12 and 24 h (*P* < 0.05)
Helmond et al. (164)	Acute postoperative pain	RCT	COX-2 inhibition (60); placebo (58)	PVB + parecoxib 80 mg on day of surgery, thereafter celecoxib 400 mg daily until postoperative day (POD) 5	PVB + placebo	VAS POD 1,5,15, and mo 1,3,6, & 12 (secondary outcome)	COX-2 inhibition only had an effect on pain on movement at POD 5 (*p* < 0.01)
Ilfield et al. (165)	Acute postoperative pain	RCT	Cryoneurolysis (43); sham (41)	Paravertebral catheter + cryoneurolysis of ipsilateral T2-T5 intercostal nerves	Paravertebral catheter + sham	Pain on numeric rating scale (NRS) on POD 2 (primary outcome). Participants followed for 1 year	POD 2 cryoneurolysis vs. sham had −2.5 (97.5% CI −3.5 to −1.5) difference in NRS vs. sham. Evidence of superior analgesia through 12 months
Tasmuth et al. (166)	Postoperative neuropathic pain after oncological treatment (chemo-, radio-, & hormonal therapy)	Crossover	13	Venlafaxine 18.75 mg tablets taken daily, increased by one at 1 week intervals for 4 weeks	Placebo	Average daily pain intensity by diary	No significant differences
Abbas et al. (167)	Post-mastectomy pain syndrome (PMPS)	RCT	Pulsed radiofrequency (RF) (52); thermal RF (52)	Thermal RF of stellate ganglion	Super voltage pulsed RF	VAS pain scores at 1,4,12, and 24 weeks (primary outcome)	Thermal RF group scores were significantly higher vs. pulsed RF group at each time point

BPI, brief pain inventory; ENS, eastern cooperative oncology group neuropathy scale; FACT-Ntx, functional assessment of cancer treatment neurotoxicity; GM1, ganglioside-monosialic acid; HT, healing touch; IV, intravenously; LA, local anesthetic; MT, massage therapy; NRS, numeric rating scale; P, caring presence; P-APS, paclitaxel-induced acute pain syndrome; PEC, pec blocks I & II; PMPS, post-mastectomy pain syndrome; POD, postoperative day; PVB, paravertebral block; RCT, randomized controlled trial; RF, radiofrequency; SA, social attention; SPB, serratus plane block; VAS, visual analog scale.

For treatment of acute surgical pain, standard regimen includes nonsteroidal anti-inflammatory drugs (NSAIDs), acetaminophen, and opioids. Systemic dexamethasone has also been demonstrated to positively impact post-operative pain, nausea, and vomiting in patients who underwent lumpectomy and mastectomy procedures (145, 146). An induction of 0.25 mg/kg followed by an IV infusion of 2–10 mcg/kg/min of ketamine administered intraoperatively has also been effective in significantly reducing the incidence of acute post-operative pain in comparison to intraoperative opioids used during breast cancer surgery (147).

Local nerve blocks represent another mode of anesthesia commonly used in breast surgeries. Multiple studies have demonstrated their ability to reduce acute post-surgical pain (148, 168, 169). In addition to acute pain, local nerve blocks have been shown to have some ability to mitigate longer-lasting post-operative pain as well, with results ranging from a few months to a year (148, 149). One study also found that receiving a paravertebral nerve block lowered average doses of narcotics taken by mastectomy patients over a six-month post-operative period (170). While there are several types of local nerve blocks, comparative studies have found that paravertebral nerve blocks seem to be the most impactful in terms of analgesia efficacy and duration (162, 163).

Studies have also evaluated the efficacy of postoperative analgesics for pain control after oncologic breast surgery. One study revealed that postoperative COX-2 inhibitors in conjunction with paravertebral neve blocks decreased pain intensity with activity on post-op day five but demonstrated no effect on postoperative hyperalgesia (164). Ultimately, there is significant variation in pain management strategies after breast surgery and often involves multimodal approach including regional nerve blocks, narcotics, and non-opioid medication use (171). Meta-analysis has also demonstrated that acupuncture is effective for surgery-induced pain (151).

Furthermore, some studies have demonstrated the efficacy of pre-operative gabapentin and pregabalin for the treatment of acute and chronic post-surgical pain after breast cancer surgery in terms of both reduced pain scores and opioid use in the post-operative recovery area (150). However, there is limited literature to substantiate these findings, and further investigation is necessary to confirm the efficacy of pre-emptive analgesic therapies.

Efforts are being made to establish interventions that decrease the risk of PMPS as well. For example, cryotherapy is being studied as a well-tolerated intervention that may prevent post-mastectomy chronic pain (165). A recent randomized control trial treated 31 participants undergoing mastectomy with an ultrasound-guided percutaneous cryoneurolysis procedure and 29 participants with a sham procedure (165). On post-operative day 2, these participants had a median pain score of 0, significantly lower than the median pain score of 3.0 for their sham-procedure counterparts [difference −2.5 (97.5% CI, −3.5 to −1.5)] (165). This improved analgesia lasted throughout a year, with chronic pain developing in 3% of cryoneurolysis participants compared to 17% of sham participants at the one-year checkpoint (165).

The literature for management of PMPS is not as extensive as that for acute post-operative pain. Currently, the management of PMPS is multidisciplinary (172). Randomized clinical trial results support the potential of medical management of PMPS including pregabalin or venlafaxine, which have been demonstrated to result in significant reductions in pain (166, 173). For pain intractable to such pharmacologic interventions, one study has shown that pulsed radiofrequency of the stellate ganglion can successfully reduce neuropathic pain intensity and increase functional improvement for up to 6 months (167). Ultrasound-guided peripheral nerve blocks have also been described for treatment of PMPS (149). Furthermore, there are situations in which surgical interventions are warranted, particularly in the case of peripheral nerve injury. A retrospective study found that 16.5 months after intercostal nerve resection and implantation, 6 of the 10 patients self-reported excellent results, and an additional patient reported good results (174).

## 7. Discussion

Breast cancer pain is multifactorial (Table 1). Pain can come from the cancer itself and may arise from treatment-related factors. Pain is also influenced by patient-specific factors, such as socioeconomic factors, psychological factors, and medical comorbidities. Though recent clinical trials have focused their efforts on the reduction and relief of acute postoperative pain, as well as chemo-, radio-, and hormonal therapy induced pain, treatment of cancer-related pain often relies on opioids, and there is need for further study of effective and safe pain therapies.

Pain management research needs to include interventions for the prevention of PMPS, for established PMPS, and other chronic pain caused by breast cancer treatments. The authors are currently studying perioperative ketamine for the prevention of PMPS (NCT05037123), hypothesizing that by targeting acute postoperative pain and mood, the severity and incidence of PMPS will be decreased, overall reducing the use of long-term opioid use, as well as reducing the risk of chronic postoperative pain in women with breast cancer.

## References

[1] Key Statistics for Breast Cancer American Cancer Society. [updated January 12, 2023]. Available at: https://www.cancer.org/cancer/breast-cancer/about/how-common-is-breast-cancer.html

[2] Lukasiewicz S Czeczelewski M Forms A Baj J Sitarz R Stanislawek A. Breast cancer-epidemiology, risk factors, classification, prognostic markers, and current treatment strategies-an updated review. Cancers (Basel). (2021) 13(17):4287. 10.3390/cancers13174287 34503097 PMC8428369

[3] DeSantis CE Fedewa SA Goding Sauer A Kramer JL Smith RA Jemal A. Breast cancer statistics, 2015: convergence of incidence rates between black and white women. CA Cancer J Clin. (2016) 66(1):31–42. 10.3322/caac.21320 26513636

[4] Sung H Ferlay J Siegel RL Laversanne M Soerjomataram I Jemal A Global cancer statistics 2020: GLOBOCAN estimates of incidence and mortality worldwide for 36 cancers in 185 countries. CACancer J Clin. (2021) 71(3):209–49. 10.3322/caac.21660 33538338

[5] Duggan C Dvaladze A Rositch AF Ginsburg O Yip CH Horton S The breast health global initiative 2018 global summit on improving breast healthcare through resource-stratified phased implementation: methods and overview. Cancer. (2020) 126(Suppl 10):2339–52. 10.1002/cncr.32891 32348573 PMC7482869

[6] Ferlay JL Laversanne M Ervik M Lam F Colombet M Mery L Global cancer observatory: Cancer tomorrow. Lyon, France: International Agency for Research on Cancer (2020). Available at: https://gco.iarc.fr/tomorrow/en.

[7] Ramani PA Niharika VS Lakhsmi BKM Jahnavi S Reddy GVS. Incidence of locally advanced breast cancer in women presenting to a tertiary care center. Int J Surg. (2019) 6(10):3626–31. 10.18203/2349-2902.isj20194415

[8] Addington J Freimer M. Chemotherapy-induced peripheral neuropathy: an update on the current understanding. F1000Res. (2016) 5:1466. 10.12688/f1000research.8053.1 PMC4920214 27408692

[9] Asthana R Zhang L Wan BA Gallo-Hershberg D Giotis A Pasetka M Pain descriptors of taxane acute pain syndrome (TAPS) in breast cancer patients-a prospective clinical study. Support Care Cancer. (2020) 28(2):589–98. 10.1007/s00520-019-04845-7 31098795

[10] Hellerstedt-Borjesson S Nordin K Fjallskog ML Peterson M Arving C. Taxane-induced pain in breast cancer patients as perceived by nurses. Acta Oncol. (2021) 60(4):412–8. 10.1080/0284186X.2021.1881816 33567934

[11] Fernandes R Mazzarello S Hutton B Shorr R Majeed H Ibrahim MF Taxane acute pain syndrome (TAPS) in patients receiving taxane-based chemotherapy for breast cancer-a systematic review. Support Care Cancer. (2016) 24(8):3633–50. 10.1007/s00520-016-3256-5 27146496

[12] Chiu N Zhang L Dent R Giotis A van Draanen J Gallo-Hershberg D A prospective study of docetaxel-associated pain syndrome. Support Care Cancer. (2018) 26(1):203–11. 10.1007/s00520-017-3836-z 28733699

[13] Gumusay O Callan J Rugo H. Immunotherapy toxicity: identification and management. Breast Cancer Res Treat. (2022) 192(1):1–17. 10.1007/s10549-021-06480-5 35015209 PMC8841316

[14] Lam E Wong G Zhang L Drost L Karam I Yee C Self-reported pain in breast cancer patients receiving adjuvant radiotherapy. Support Care Cancer. (2021) 29(1):155–67. 10.1007/s00520-020-05462-5 32323002

[15] McQuestion M. Evidence-based skin care management in radiation therapy: clinical update. Semin Oncol Nurs. (2011) 27(2):e1–17. 10.1016/j.soncn.2011.02.009 21514477

[16] Costa WA Monteiro MN Queiroz JF Gonçalves AK. Pain and quality of life in breast cancer patients. Clinics. (2017) 72:758–63. 10.6061/clinics/2017(12)07 29319722 PMC5738557

[17] Andersen KG Kehlet H. Persistent pain after breast cancer treatment: a critical review of risk factors and strategies for prevention. J Pain. (2011) 12(7):725–46. 10.1016/j.jpain.2010.12.005 21435953

[18] Jung BF Ahrendt GM Oaklander AL Dworkin RH. Neuropathic pain following breast cancer surgery: proposed classification and research update. Pain. (2003) 104(1–2):1–13. 10.1016/s0304-3959(03)00241-0 12855309

[19] Kehlet H Jensen TS Woolf CJ. Persistent postsurgical pain: risk factors and prevention. Lancet. (2006) 367(9522):1618–25. 10.1016/S0140-6736(06)68700-X 16698416

[20] Glare PA Davies PS Finlay E Gulati A Lemanne D Moryl N Pain in cancer survivors. J Clin Oncol. (2014) 32(16):1739–47. 10.1200/JCO.2013.52.4629 24799477 PMC4031191

[21] Bao T Seidman A Li Q Seluzicki C Blinder V Meghani SH Living with chronic pain: perceptions of breast cancer survivors. Breast Cancer Res Treat. (2018) 169(1):133–40. 10.1007/s10549-018-4670-9 29350307 PMC6147242

[22] Vilholm OJ Cold S Rasmussen L Sindrup SH. The postmastectomy pain syndrome: an epidemiological study on the prevalence of chronic pain after surgery for breast cancer. Br J Cancer. (2008) 99(4):604–10. 10.1038/sj.bjc.6604534 18682712 PMC2527825

[23] Caffo O Amichetti M Ferro A Lucenti A Valduga F Galligioni E. Pain and quality of life after surgery for breast cancer. Breast Cancer Res Treat. (2003) 80(1):39–48. 10.1023/A:1024435101619 12889597

[24] Gartner R Jensen MB Nielsen J Ewertz M Kroman N Kehlet H. Prevalence of and factors associated with persistent pain following breast cancer surgery. JAMA. (2009) 302(18):1985–92. 10.1001/jama.2009.1568 19903919

[25] Peuckmann V Ekholm O Rasmussen NK Groenvold M Christiansen P Moller S Chronic pain and other sequelae in long-term breast cancer survivors: nationwide survey in Denmark. Eur J Pain. (2009) 13(5):478–85. 10.1016/j.ejpain.2008.05.015 18635381

[26] Smith WC Bourne D Squair J Phillips DO Chambers WA. A retrospective cohort study of post mastectomy pain syndrome. Pain. (1999) 83(1):91–5. 10.1016/s0304-3959(99)00076-7 10506676

[27] Poleshuck EL Katz J Andrus CH Hogan LA Jung BF Kulick DI Risk factors for chronic pain following breast cancer surgery: a prospective study. J Pain. (2006) 7(9):626–34. 10.1016/j.jpain.2006.02.007 16942948 PMC6983301

[28] Leysen L Beckwee D Nijs J Pas R Bilterys T Vermeir S Risk factors of pain in breast cancer survivors: a systematic review and meta-analysis. Support Care Cancer. (2017) 25(12):3607–43. 10.1007/s00520-017-3824-3 28799015

[29] Extermann M. Measurement and impact of comorbidity in older cancer patients. Crit Rev Oncol Hematol. (2000) 35(3):181–200. 10.1016/s1040-8428(00)00090-1 10960800

[30] Schou Bredal I Smeby NA Ottesen S Warncke T Schlichting E. Chronic pain in breast cancer survivors: comparison of psychosocial, surgical, and medical characteristics between survivors with and without pain. J Pain Symptom Manage. (2014) 48(5):852–62. 10.1016/j.jpainsymman.2013.12.239 24703940

[31] Johannsen M Christensen S Zachariae R Jensen AB. Socio-demographic, treatment-related, and health behavioral predictors of persistent pain 15 months and 7–9 years after surgery: a nationwide prospective study of women treated for primary breast cancer. Breast Cancer Res Treat. (2015) 152(3):645–58. 10.1007/s10549-015-3497-x 26189085

[32] Alkan A Guc ZG Senler FC Yavuzsen T Onur H Dogan M Breast cancer survivors suffer from persistent postmastectomy pain syndrome and posttraumatic stress disorder (ORTHUS study): a study of the palliative care working committee of the Turkish oncology group (TOG). Support Care Cancer. (2016) 24(9):3747–55. 10.1007/s00520-016-3202-6 27039206

[33] Bell RJ Robinson PJ Nazeem F Panjari M Fradkin P Schwarz M Persistent breast pain 5 years after treatment of invasive breast cancer is largely unexplained by factors associated with treatment. J Cancer Surviv. (2014) 8(1):1–8. 10.1007/s11764-013-0306-6 23975613

[34] Storey S Cohee A Gathirua-Mwangi WG Vachon E Monahan P Otte J The impact of diabetes on the symptoms of breast cancer survivors. Oncol Nurs Forum. (2019) 46(4):473–84. 10.1188/19.ONF.473-484 31225841 PMC6936108

[35] Coughlin SS Ayyala D. Symptoms associated with comorbid diabetes among breast cancer survivors. Breast Cancer Res Treat. (2021) 189:781–6. 10.1007/s10549-021-06324-2 34244868

[36] Yang GS Barnes NM Lyon DE Dorsey SG. Genetic variants associated with cancer pain and response to opioid analgesics: implications for precision pain management. Semin Oncol Nurs. (2019) 35(3):291–9. 10.1016/j.soncn.2019.04.011 31085105 PMC6688486

[37] McCann B Miaskowski C Koetters T Baggott C West C Levine JD Associations between pro- and anti-inflammatory cytokine genes and breast pain in women prior to breast cancer surgery. J Pain. (2012) 13(5):425–37. 10.1016/j.jpain.2011.02.358 22515947 PMC3348353

[38] Stephens KE Levine JD Aouizerat BE Paul SM Abrams G Conley YP Associations between genetic and epigenetic variations in cytokine genes and mild persistent breast pain in women following breast cancer surgery. Cytokine. (2017) 99:203–13. 10.1016/j.cyto.2017.07.006 28764974 PMC5675785

[39] Torres R Macdonald L Croll SD Reinhardt J Dore A Stevens S Hyperalgesia, synovitis and multiple biomarkers of inflammation are suppressed by interleukin 1 inhibition in a novel animal model of gouty arthritis. Ann Rheum Dis. (2009) 68(10):1602–8. 10.1136/ard.2009.109355 19528034

[40] Moloney NA Pocovi NC Dylke ES Graham PL De Groef A. Psychological factors are associated with pain at all time frames after breast cancer surgery: a systematic review with meta-analyses. Pain Med. (2021) 22(4):915–47. 10.1093/pm/pnaa363 33547465

[41] Bair MJ Robinson RL Katon W Kroenke K. Depression and pain comorbidity: a literature review. Arch Intern Med. (2003) 163(20):2433–45. 10.1001/archinte.163.20.2433 14609780

[42] Katz J Poleshuck EL Andrus CH Hogan LA Jung BF Kulick DI Risk factors for acute pain and its persistence following breast cancer surgery. Pain. (2005) 119(1–3):16–25. 10.1016/j.pain.2005.09.008 16298063

[43] Boersma K Linton SJ. Screening to identify patients at risk: profiles of psychological risk factors for early intervention. Clin J Pain. (2005) 21(1):38–43; discussion 69–72. 10.1097/00002508-200501000-00005 15599130

[44] Rashiq S Dick BD. Post-surgical pain syndromes: a review for the non-pain specialist. Can J Anaesth. (2014) 61(2):123–30. 10.1007/s12630-013-0072-y 24185829

[45] Hirsh AT George SZ Bialosky JE Robinson ME. Fear of pain, pain catastrophizing, and acute pain perception: relative prediction and timing of assessment. J Pain. (2008) 9(9):806–12. 10.1016/j.jpain.2008.03.012 18486557 PMC2568976

[46] Forsythe LP Alfano CM George SM McTiernan A Baumgartner KB Bernstein L Pain in long term breast cancer survivors: the role of body mass index, physical activity, and sedentary behavior. Breast Cancer Res Treat. (2013) 137(2):617–30. 10.1007/s10549-012-2335-7 23242613 PMC3992941

[47] Moloney N Sung JM Kilbreath S Dylke E. Prevalence and risk factors associated with pain 21 months following surgery for breast cancer. Support Care Cancer. (2016) 24(11):4533–9. 10.1007/s00520-016-3292-1 27271868

[48] Alfano CM Smith AW Irwin ML Bowen DJ Sorensen B Reeve BB Physical activity, long-term symptoms, and physical health-related quality of life among breast cancer survivors: a prospective analysis. J Cancer Surviv. (2007) 1(2):116–28. 10.1007/s11764-007-0014-1 18648952 PMC2996230

[49] Rief W Bardwell WA Dimsdale JE Natarajan L Flatt SW Pierce JP. Long-term course of pain in breast cancer survivors: a 4-year longitudinal study. Breast Cancer Res Treat. (2011) 130(2):579–86. 10.1007/s10549-011-1614-z 21656272 PMC3681533

[50] Sipila R Estlander AM Tasmuth T Kataja M Kalso E. Development of a screening instrument for risk factors of persistent pain after breast cancer surgery. Br J Cancer. (2012) 107(9):1459–66. 10.1038/bjc.2012.445 23093294 PMC3493779

[51] Reis AD Pereira P Diniz RR de Castro Filha JGL Dos Santos AM Ramallo BT Effect of exercise on pain and functional capacity in breast cancer patients. Health Qual Life Outcomes. (2018) 16(1):58. 10.1186/s12955-018-0882-2 29625622 PMC5889570

[52] Panis C Pavanelli WR. Cytokines as mediators of pain-related process in breast cancer. Mediators Inflamm. (2015) 2015:129034. 10.1155/2015/129034 26635447 PMC4655288

[53] Satija A Ahmed SM Gupta R Ahmed A Rana SP Singh SP Breast cancer pain management—a review of current & novel therapies. Indian J Med Res. (2014) 139(2):216–25. 24718395 PMC4001332

[54] Anampa J Makower D Sparano JA. Progress in adjuvant chemotherapy for breast cancer: an overview. BMC Med. (2015) 13:195. 10.1186/s12916-015-0439-8 26278220 PMC4538915

[55] Fuster D Herranz D Vidal-Sicart S Munoz M Conill C Mateos JJ Usefulness of strontium-89 for bone pain palliation in metastatic breast cancer patients. Nucl Med Commun. (2000) 21(7):623–6. 10.1097/00006231-200007000-00004 10994664

[56] Stubblefield MD Custodio CM. Upper-extremity pain disorders in breast cancer. Arch Phys Med Rehabil. (2006) 87(3 Suppl 1):S96–29; quiz S100–1. 10.1016/j.apmr.2005.12.017 16500198

[57] Manders K van de Poll-Franse LV Creemers GJ Vreugdenhil G van der Sangen MJ Nieuwenhuijzen GA Clinical management of women with metastatic breast cancer: a descriptive study according to age group. BMC Cancer. (2006) 6:179. 10.1186/1471-2407-6-179 16824210 PMC1534056

[58] Kennecke H Yerushalmi R Woods R Cheang MC Voduc D Speers CH Metastatic behavior of breast cancer subtypes. J Clin Oncol. (2010) 28(20):3271–7. 10.1200/JCO.2009.25.9820 20498394

[59] Roodman GD. Mechanisms of bone metastasis. N Engl J Med. (2004) 350(16):1655–64. 10.1056/NEJMra030831 15084698

[60] Chirgwin JM Guise TA. Molecular mechanisms of tumor-bone interactions in osteolytic metastases. Crit Rev Eukaryot Gene Expr. (2000) 10(2):159–78. 10.1615/CritRevEukarGeneExpr.v10.i2.50 11186331

[61] Tahara RK Brewer TM Theriault RL Ueno NT. Bone metastasis of breast cancer. Adv Exp Med Biol. (2019) 1152:105–29. 10.1007/978-3-030-20301-6_7 31456182

[62] Oldenmenger WH Geerling JI Mostovaya I Vissers KCP de Graeff A Reyners AKL A systematic review of the effectiveness of patient-based educational interventions to improve cancer-related pain. Cancer Treat Rev. (2018) 63:96–103. 10.1016/j.ctrv.2017.12.005 29272781

[63] Sugimoto H Oda G Yokoyama M Hayashi K Yoshino M Ogawa A Hydronephrosis caused by metastatic breast cancer. Case Rep Oncol. (2021) 14(1):378–85. 10.1159/000513903 33776732 PMC7983561

[64] Berry DA Cronin KA Plevritis SK Fryback DG Clarke L Zelen M Effect of screening and adjuvant therapy on mortality from breast cancer. N Engl J Med. (2005) 353(17):1784–92. 10.1056/NEJMoa050518 16251534

[65] Romero A Tora-Rocamora I Bare M Barata T Domingo L Ferrer J Prevalence of persistent pain after breast cancer treatment by detection mode among participants in population-based screening programs. BMC Cancer. (2016) 16(1):735. 10.1186/s12885-016-2768-1 27632982 PMC5025583

[66] Gulluoglu BM Cingi A Cakir T Gercek A Barlas A Eti Z. Factors related to post-treatment chronic pain in breast cancer survivors: the interference of pain with life functions. Int J Fertil Womens Med. (2006) 51(2):75–82. 16881383

[67] Grisold W Cavaletti G Windebank AJ. Peripheral neuropathies from chemotherapeutics and targeted agents: diagnosis, treatment, and prevention. Neuro Oncol. (2012) 14(Suppl 4):iv45–54. 10.1093/neuonc/nos203 23095830 PMC3480245

[68] Society AC. *Treating breast cancer 2021* [updated 10/27/2021]. Available at: https://www.cancer.org/content/dam/CRC/PDF/Public/8581.00.pdf

[69] Fenlon D Addington-Hall JM O'Callaghan AC Clough J Nicholls P Simmonds P. A survey of joint and muscle aches, pain, and stiffness comparing women with and without breast cancer. J Pain Symptom Manage. (2013) 46(4):523–35. 10.1016/j.jpainsymman.2012.10.282 23507130

[70] Chaudhry V Chaudhry M Crawford TO Simmons-O'Brien E Griffin JW. Toxic neuropathy in patients with pre-existing neuropathy. Neurology. (2003) 60(2):337–40. 10.1212/01.wnl.0000043691.53710.53 12552058

[71] Eckhoff L Knoop A Jensen MB Ewertz M. Persistence of docetaxel-induced neuropathy and impact on quality of life among breast cancer survivors. Eur J Cancer. (2015) 51(3):292–300. 10.1016/j.ejca.2014.11.024 25541155

[72] Seguin C Kovacevich N Voutsadakis IA. Docetaxel-associated myalgia–arthralgia syndrome in patients with breast cancer. Breast Cancer (Dove Med Press). (2017) 9:39–44. 10.2147/BCTT.S124646 28176956 PMC5271389

[73] Haba-Rodriguez J Rodríguez-Lescure A Ruiz A Alba E Calvo L Carrasco E Regional and seasonal influence in patient’s toxicity to adjuvant chemotherapy for early breast cancer. Breast Cancer Res Treat. (2011) 125(1):273–8. 10.1007/s10549-010-1136-0 20803065

[74] Wist EA Mjaaland I Lokkevik E Sommer HH. Weekly paclitaxel plus capecitabine versus docetaxel every 3 weeks plus capecitabine in metastatic breast cancer. J Oncol. (2012) 2012:862921. 10.1155/2012/862921 22291703 PMC3265117

[75] Luck HJ Du Bois A Loibl S. Capecitabine plus paclitaxel versus epirubicin plus paclitaxel as first-line treatment for metastatic breast cancer: efficacy and safety results of a randomized, phase III trial by the AGO breast cancer study group. Breast Cancer Res Treat. (2013) 139(3):779–87. 10.1007/s10549-013-2589-8 23771714

[76] Christensen JF Jones LW Andersen JL Daugaard G Rorth M Hojman P. Muscle dysfunction in cancer patients. Ann Oncol. (2014) 25(5):947–58. 10.1093/annonc/mdt551 24401927

[77] Ballinger TJ Reddy A Althouse SK Nelson EM Miller KD Sledge JS. Impact of primary breast cancer therapy on energetic capacity and body composition. Breast Cancer Res Treat. (2018) 172(2):445–52. 10.1007/s10549-018-4924-6 30136009 PMC6208924

[78] Freedman RJ Aziz N Albanes D Hartman T Danforth D Hill S Weight and body composition changes during and after adjuvant chemotherapy in women with breast cancer. J Clin Endocrinol Metab. (2004) 89(5):2248–53. 10.1210/jc.2003-031874 15126549

[79] Gatti-Mays ME Balko JM Gameiro SR Bear HD Prabhakaran S Fukui J If we build it they will come: targeting the immune response to breast cancer. NPJ Breast Cancer. (2019) 5:37. 10.1038/s41523-019-0133-7 31700993 PMC6820540

[80] Barzaman K Moradi-Kalbolandi S Hosseinzadeh A Kazemi MH Khorramdelazad H Safari E Breast cancer immunotherapy: current and novel approaches. Int Immunopharmacol. (2021) 98:107886. 10.1016/j.intimp.2021.107886 34153663

[81] Jahanzeb M. Adjuvant trastuzumab therapy for HER2-positive breast cancer. Clin Breast Cancer. (2008) 8(4):324–33. 10.3816/CBC.2008.n.037 18757259

[82] Dillman RO. Infusion reactions associated with the therapeutic use of monoclonal antibodies in the treatment of malignancy. Cancer Metastasis Rev. (1999) 18(4):465–71. 10.1023/a:1006341717398 10855789

[83] Goldenberg MM. Trastuzumab, a recombinant DNA-derived humanized monoclonal antibody, a novel agent for the treatment of metastatic breast cancer. Clin Ther. (1999) 21(2):309–18. 10.1016/S0149-2918(00)88288-0 10211534

[84] Cook-Bruns N. Retrospective analysis of the safety of herceptin immunotherapy in metastatic breast cancer. Oncology. (2001) 61(Suppl 2):58–66. 10.1159/000055403 11694789

[85] Schmid P Rugo HS Adams S Schneeweiss A Barrios CH Iwata H Atezolizumab plus nab-paclitaxel as first-line treatment for unresectable, locally advanced or metastatic triple-negative breast cancer (IMpassion130): updated efficacy results from a randomised, double-blind, placebo-controlled, phase 3 trial. Lancet Oncol. (2020) 21(1):44–59. 10.1016/S1470-2045(19)30689-8 31786121

[86] Criscitiello C Corti C Pravettoni G Curigliano G. Managing side effects of immune checkpoint inhibitors in breast cancer. Crit Rev Oncol Hematol. (2021) 162:103354. 10.1016/j.critrevonc.2021.103354 34029683

[87] Haanen J Carbonnel F Robert C Kerr KM Peters S Larkin J Management of toxicities from immunotherapy: ESMO clinical practice guidelines for diagnosis, treatment and follow-up. Ann Oncol. (2017) 28(suppl_4):iv119–42. 10.1093/annonc/mdx225 28881921

[88] Krishnadas DK Shusterman S Bai F Diller L Sullivan JE Cheerva AC A phase I trial combining decitabine/dendritic cell vaccine targeting MAGE-A1, MAGE-A3 and NY-ESO-1 for children with relapsed or therapy-refractory neuroblastoma and sarcoma. Cancer Immunol Immunother. (2015) 64(10):1251–60. 10.1007/s00262-015-1731-3 26105625 PMC11028635

[89] Mitchell DA Sayour EJ Reap E Schmittling R DeLeon G Norberg P Severe adverse immunologic reaction in a patient with glioblastoma receiving autologous dendritic cell vaccines combined with GM-CSF and dose-intensified temozolomide. Cancer Immunol Res. (2015) 3(4):320–5. 10.1158/2326-6066.CIR-14-0100 25387895 PMC4510873

[90] European Medicines Agency. *Product information herceptin (trastuzumab)*. (2017). Available at: http://www.ema.europa.eu/docs/en_GB/document_library/EPAR_-_Product_Information/human/000278/WC500074922.pdf

[91] Williams TH Benavides B Patrice K Dalmau J de Avila A Le D Association of autoimmune encephalitis with combined immune checkpoint inhibitor treatment for metastatic cancer. JAMA Neurol. (2016) 73(8):923–33. 10.1001/jamaneurol.2016.1399 27271951

[92] Chung M Jaffer M Verma N Mokhtari S Ramsakal A Peguero E. Immune checkpoint inhibitor induced anti-glutamic acid decarboxylase 65 (anti-GAD 65) limbic encephalitis responsive to intravenous immunoglobulin and plasma exchange. J Neurol. (2019) 267(4):1023–5. 10.1007/s00415-019-09666-6 31832829

[93] Matsuda M Huh Y Ji R. Roles of inflammation, neurogenic inflammation, and neuroinflammation in pain. J Anesth. (2019) 33:131–9. 10.1007/s00540-018-2579-4 30448975 PMC6813778

[94] Tang NKY Salkovskis P Hodges A Weight KJ Hanna M Hester J. Effects of mood on pain responses and pain tolerance: an experimental study in chronic back pain patients. Pain. (2008) 138(2):392–401. 10.1016/j.pain.2008.01.018 18325674

[95] Delanian S Lefaix JL. The radiation-induced fibroatrophic process: therapeutic perspective via the antioxidant pathway. Radiother Oncol. (2004) 73(2):119–31. 10.1016/j.radonc.2004.08.021 15542158

[96] Delanian S Lefaix JL Pradat PF. Radiation-induced neuropathy in cancer survivors. Radiother Oncol. (2012) 105(3):273–82. 10.1016/j.radonc.2012.10.012 23245644

[97] Wang L Guyatt GH Kennedy SA Romerosa B Kwon HY Kaushal A Predictors of persistent pain after breast cancer surgery: a systematic review and meta-analysis of observational studies. CMAJ. (2016) 188(14):E352–E61. 10.1503/cmaj.151276 27402075 PMC5047835

[98] Warade AC Jha AK Pattankar S Desai K. Radiation-induced brachial plexus neuropathy: a review. Neurol India. (2019) 67(Supplement):S47–52. 10.4103/0028-3886.250704 30688233

[99] Drost L Li N Vesprini D Sangha A Lee J Leung E Prospective study of breast radiation dermatitis. Clin Breast Cancer. (2018) 18(5):e789–95. 10.1016/j.clbc.2018.03.008 29622382

[100] Barchiesi G Mazzotta M Krasniqi E Pizzuti L Marinelli D Capomolla E Neoadjuvant endocrine therapy in breast cancer: current knowledge and future perspectives. Int J Mol Sci. (2020) 21(10):3528. 10.3390/ijms21103528 32429381 PMC7278946

[101] Jung BF Herrmann D Griggs J Oaklander AL Dworkin RH. Neuropathic pain associated with non surgical treatment of breast cancer. Pain. (2005) 118(1–2):10–4. 10.1016/j.pain.2005.09.014 16213086

[102] Plotkin SR Wen PY. Neurologic complications of cancer therapy. Neurol Clin. (2003) 21(1):279–318, x. 10.1016/s0733-8619(02)00034-8 12690653

[103] Johansen J Overgaard J Overgaard M. Effect of adjuvant systemic treatment on cosmetic outcome and late normal-tissue reactions after breast conservation. Acta Oncol. (2007) 46(4):525–33. 10.1080/02841860701291698 17497320

[104] Gaillard S Stearns V. Aromatase inhibitor-associated bone and musculoskeletal effects: new evidence defining etiology and strategies for management. Breast Cancer Res. (2011) 13(2):205. 10.1186/bcr2818 21457526 PMC3219175

[105] Mak KS Chen YH Catalano PJ Punglia RS Wong JS Truong L Dosimetric inhomogeneity predicts for long-term breast pain after breast-conserving therapy. Int J Radiat Oncol Biol Phys. (2015) 93(5):1087–95. 10.1016/j.ijrobp.2014.05.021 25084611

[106] Younus J Kligman L. Management of aromatase inhibitor-induced arthralgia. Curr Oncol. (2010) 17(1):87–90. 10.3747/co.v17i1.474 20179809 PMC2826784

[107] Mishra G Kuh D. Perceived change in quality of life during the menopause. Soc Sci Med. (2006) 62(1):93–102. 10.1016/j.socscimed.2005.05.015 15990213

[108] Buzdar AU. Clinical features of joint symptoms observed in the “arimidex”, tamoxifen, alone or in combination (ATAC) trial. J Clin Oncol. (2006) 24(18_suppl):551. 10.1200/jco.2006.24.18_suppl.551

[109] Briot K Tubiana-Hulin M Bastit L Kloos I Roux C. Effect of a switch of aromatase inhibitors on musculoskeletal symptoms in postmenopausal women with hormone-receptor-positive breast cancer: the ATOLL (articular tolerance of letrozole) study. Breast Cancer Res Treat. (2010) 120(1):127–34. 10.1007/s10549-009-0692-7 20035381

[110] Wright CL Mist S Ross RL Jones KD. Duloxetine for the treatment of fibromyalgia. Expert Rev Clin Immunol. (2010) 6(5):745–56. 10.1586/eci.10.64 20828282 PMC3056054

[111] Bril V England J Franklin GM Backonja M Cohen J Del Toro D Feldman E Evidence-based guideline: treatment of painful diabetic neuropathy—report of the American association of neuromuscular and electrodiagnostic medicine, the American academy of neurology, and the American academy of physical medicine & rehabilitation. Muscle Nerve. (2011) 46(3):910–7. 10.1002/mus.22092 21484835

[112] Anand KS Dhikav V Prasad A Shewtengna. Efficacy, safety and tolerability of duloxetine in idiopathic trigeminal neuralgia. J Indian Med Assoc. (2011) 109(4):264–6. . 22187799

[113] Tsuda M Toi M. Duloxetine for AI-associated joint pain in breast cancer patients. Transl Cancer Res. (2018) 7(36):326–32. 10.21037/tcr.2018.03.28

[114] Chow R Novosel M So OW Bellampalli S Xiang J Boldt G Duloxetine for prevention and treatment of chemotherapy-induced peripheral neuropathy (CIPN): systematic review and meta-analysis. BMJ Support Palliat Care. (2023) 13(1):27–34. 10.1136/spcare-2022-003815 36194493

[115] Bedrosian I Hu CY Chang GJ. Population-based study of contralateral prophylactic mastectomy and survival outcomes of breast cancer patients. J Natl Cancer Inst. (2010) 102(6):401–9. 10.1093/jnci/djq018 20185801 PMC4415082

[116] Yi M Hunt KK Arun BK Bedrosian I Barrera AG Do KA Factors affecting the decision of breast cancer patients to undergo contralateral prophylactic mastectomy. Cancer Prev Res (Phila). (2010) 3(8):1026–34. 10.1158/1940-6207.CAPR-09-0130 20647335 PMC4340054

[117] Andersen KG Duriaud HM Aasvang EK Kehlet H. Association between sensory dysfunction and pain 1 week after breast cancer surgery: a psychophysical study. Acta Anaesthesiol Scand. (2016) 60(2):259–69. 10.1111/aas.12641 26446738

[118] Gassman AA Yoon AP Festekjian J Da Lio AL Tseng CY Crisera C. Comparison of immediate postoperative pain in implant-based breast reconstructions. J Plast Reconstr Aesthet Surg. (2016) 69(5):604–16. 10.1016/j.bjps.2015.12.009 26947947

[119] Kulkarni AR Pusic AL Hamill JB Kim HM Qi J Wilkins EG Factors associated with acute postoperative pain following breast reconstruction. JPRAS Open. (2017) 11:1–13. 10.1016/j.jpra.2016.08.005 28713853 PMC5507622

[120] Schreiber KL Zinboonyahgoon N Xu X Spivey T King T Dominici L Preoperative psychosocial and psychophysical phenotypes as predictors of acute pain outcomes after breast surgery. J Pain. (2019) 20(5):540–56. 10.1016/j.jpain.2018.11.004 30476655 PMC6511455

[121] Habib AS Kertai MD Cooter M Greenup RA Hwang S. Risk factors for severe acute pain and persistent pain after surgery for breast cancer: a prospective observational study. Reg Anesth Pain Med. (2019) 44(2):192–9. 10.1136/rapm-2018-000040 30700614

[122] Janssen KJ Kalkman CJ Grobbee DE Bonsel GJ Moons KG Vergouwe Y. The risk of severe postoperative pain: modification and validation of a clinical prediction rule. Anesth Analg. (2008) 107(4):1330–9. 10.1213/ane.0b013e31818227da 18806049

[123] Ellis A Bennett DL. Neuroinflammation and the generation of neuropathic pain. Br J Anaesth. (2013) 111(1):26–37. 10.1093/bja/aet128 23794642

[124] Lavand'homme P Steyaert A. Opioid-free anesthesia opioid side effects: tolerance and hyperalgesia. Best Pract Res Clin Anaesthesiol. (2017) 31(4):487–98. 10.1016/j.bpa.2017.05.003 29739537

[125] Werner MU Kongsgaard UE. I. Defining persistent post-surgical pain: is an update required? Br J Anaesth. (2014) 113(1):1–4. 10.1093/bja/aeu012 24554546

[126] Katz J Seltzer Z. Transition from acute to chronic postsurgical pain: risk factors and protective factors. Expert Rev Neurother. (2009) 9(5):723–44. 10.1586/ern.09.20 19402781

[127] Glare P Aubrey KR Myles PS. Transition from acute to chronic pain after surgery. Lancet. (2019) 393(10180):1537–46. 10.1016/S0140-6736(19)30352-6 30983589

[128] Althaus A Arranz Becker O Neugebauer E. Distinguishing between pain intensity and pain resolution: using acute post-surgical pain trajectories to predict chronic post-surgical pain. Eur J Pain. (2014) 18(4):513–21. 10.1002/j.1532-2149.2013.00385.x 23983024

[129] Wang K Yee C Tam S Drost L Chan S Zaki P Prevalence of pain in patients with breast cancer post-treatment: a systematic review. Breast. (2018) 42:113–27. 10.1016/j.breast.2018.08.105 30243159

[130] Rosso R Scelsi M Carnevali L. Granular cell traumatic neuroma: a lesion occurring in mastectomy scars. Arch Pathol Lab Med. (2000) 124(5):709–11. 10.5858/2000-124-0709-GCTN 10782152

[131] Woolf CJ Salter MW. Neuronal plasticity: increasing the gain in pain. Science. (2000) 288(5472):1765–9. 10.1126/science.288.5472.1765 10846153

[132] Villa G Mandarano R Scire-Calabrisotto C Rizzelli V Del Duca M Montin DP Chronic pain after breast surgery: incidence, associated factors, and impact on quality of life, an observational prospective study. Perioper Med (Lond). (2021) 10(1):6. 10.1186/s13741-021-00176-6 33622393 PMC7903732

[133] Chappell AG Bai J Yuksel S Ellis MF. Post-mastectomy pain syndrome: defining perioperative etiologies to guide new methods of prevention for plastic surgeons. World J Plast Surg. (2020) 9(3):247–53. 10.29252/wjps.9.3.247 33329999 PMC7734930

[134] Rietman JS Dijkstra PU Hoekstra HJ Eisma WH Szabo BG Groothoff JW Late morbidity after treatment of breast cancer in relation to daily activities and quality of life: a systematic review. Eur J Surg Oncol. (2003) 29(3):229–38. 10.1053/ejso.2002.1403 12657232

[135] Bromham N Schmidt-Hansen M Astin M Hasler E Reed MW. Axillary treatment for operable primary breast cancer. Cochrane Database Syst Rev. (2017) 1:CD004561. 10.1002/14651858.CD004561.pub3 28052186 PMC6464919

[136] Del Bianco P Zavagno G Burelli P Scalco G Barutta L Carraro P Morbidity comparison of sentinel lymph node biopsy versus conventional axillary lymph node dissection for breast cancer patients: results of the sentinella-GIVOM Italian randomised clinical trial. Eur J Surg Oncol. (2008) 34(5):508–13. 10.1016/j.ejso.2007.05.017 17614245

[137] Ventafridda V Stjernsward J. Pain control and the world health organization analgesic ladder. JAMA. (1996) 275(11):835–6. 10.1001/jama.1996.03530350017014 8596213

[138] Orhan ME Bilgin F Ergin A Dere K Guzeldemir ME. Pain treatment practice according to the WHO analgesic ladder in cancer patients: eight years experience of a single center. Agri. (2008) 20(4):37–43. 19117155

[139] Sloan PA. The evolving role of interventional pain management in oncology. J Support Oncol. (2004) 2(6):491–500, 3. 15605916

[140] Lutz S Berk L Chang E Chow E Hahn C Hoskin P Palliative radiotherapy for bone metastases: an ASTRO evidence-based guideline. Int J Radiat Oncol Biol Phys. (2011) 79(4):965–76. 10.1016/j.ijrobp.2010.11.026 21277118

[141] Van den Beuken-van Everdingen M De Rijke J Kessels A Schouten H Van Kleef M Patijn J. Prevalence of pain in patients with cancer: a systematic review of the past 40 years. Ann Oncol. (2007) 18(9):1437–49. 10.1093/annonc/mdm056 17355955

[142] Jane SW Chen SL Wilkie DJ Lin YC Foreman SW Beaton RD Effects of massage on pain, mood status, relaxation, and sleep in Taiwanese patients with metastatic bone pain: a randomized clinical trial. Pain. (2011) 152(10):2432–42. 10.1016/j.pain.2011.06.021 21802850

[143] Lin W Wang R Chou F Feng I Fang C Wang H. The effects of exercise on chemotherapy-induced peripheral neuropathy symptoms in cancer patients: a systematic review and meta-analysis. Support Care Cancer. (2021) 29:5303–11. 10.1007/s00520-021-06082-3 33660078

[144] Post-White J Kinney ME Savik K Gau JB Wilcox C Lerner I. Therapeutic massage and healing touch improve symptoms in cancer. Integr Cancer Ther. (2003) 2(4):332–44. 10.1177/1534735403259064 14713325

[145] Cortes-Flores AO Jimenez-Tornero J Morgan-Villela G Delgado-Gomez M Zuloaga-Fernandez Del Valle CJ Garcia-Renteria J Effects of preoperative dexamethasone on postoperative pain, nausea, vomiting and respiratory function in women undergoing conservative breast surgery for cancer: results of a controlled clinical trial. Eur J Cancer Care (Engl). (2018) 27(1):12686. 10.1111/ecc.12686 28474341

[146] Gomez-Hernandez J Orozco-Alatorre AL Dominguez-Contreras M Oceguera-Villanueva A Gomez-Romo S Alvarez Villasenor AS Preoperative dexamethasone reduces postoperative pain, nausea and vomiting following mastectomy for breast cancer. BMC Cancer. (2010) 10:692. 10.1186/1471-2407-10-692 21182781 PMC3017064

[147] Lopez M Padilla M Garcia B Orozco J Rodilla AM. Prevention of acute postoperative pain in breast cancer: a comparison between opioids versus ketamine in the intraoperatively analgesia. Pain Res Manag. (2021) 2021:3290289. 10.1155/2021/3290289 34840635 PMC8612786

[148] Kairaluoma PM Bachmann M Rosenberg PH Pere PJ. Preincisional paravertebral block reduces the prevalence of chronic pain after breast surgery. Anesth Analg. (2006) 103(3):703–8. 10.1213/01.ane.0000230603.92574.4e 16931684

[149] Yang A Nadav D Legler A Chen GH Hingula L Puttanniah V An interventional pain algorithm for the treatment of postmastectomy pain syndrome: a single-center retrospective review. Pain Med. (2021) 22(3):677–86. 10.1093/pm/pnaa343 33155049 PMC7971473

[150] Rai AS Khan JS Dhaliwal J Busse JW Choi S Devereaux PJ Preoperative pregabalin or gabapentin for acute and chronic postoperative pain among patients undergoing breast cancer surgery: a systematic review and meta-analysis of randomized controlled trials. J Plast Reconstr Aesthet Surg. (2017) 70(10):1317–28. 10.1016/j.bjps.2017.05.054 28751024

[151] Chiu HY Hsieh YJ Tsai PS. Systematic review and meta-analysis of acupuncture to reduce cancer related pain. Eur J Cancer Care (Engl). (2017) 26(2):12457. 10.1111/ecc.12457 26853524

[152] Zajaczkowska R Kocot-Kepska M Leppert W Wordliczek J. Bone pain in cancer patients: mechanisms and current treatment. Int J Mol Sci. (2019) 20(23):6047. 10.3390/ijms20236047 31801267 PMC6928918

[153] Fallon M Giusti R Aielli F Hoskin P Rolke R Sharma M Management of cancer pain in adult patients: eSMO clinical practice guidelines. Ann Oncol. (2018) 29(Suppl 4):iv166–91. 10.1093/annonc/mdy152 30052758

[154] Organization WH. WHO Guidelines for the pharmacological and radiotherapeutic management of cancer pain in adults and adolescents. Geneva: World Health Organization (2018). Available from: https://www.ncbi.nlm.nih.gov/books/NBK537492/30776210

[155] Schneider G Voltz R Gaertner J. Cancer pain management and bone metastases: an update for the clinician. Breast Care (Basel). (2012) 7(2):113–20. 10.1159/000338579 22740797 PMC3376368

[156] Singh SK Spiegel S. Sphingosine-1-phosphate signaling: a novel target for simultaneous adjuvant treatment of triple negative breast cancer and chemotherapy-induced neuropathic pain. Adv Biol Regul. (2020) 75:100670. 10.1016/j.jbior.2019.100670 31708456

[157] Su Y Huang J Wang S Unger JM Arias-Fuenzalida J Shi Y Li J The effects of ganglioside-monosialic acid in taxane-induced peripheral neurotoxicity in patients with breast cancer: a randomized trial. JNCI. (2019) 112(1):55–62. 10.1093/jnci/djz086 31093677

[158] Luo ZD Chaplan S Higuera ES Sorkin LS Stauderman KA Williams ME Upregulation of dorsal root ganglion (alpha)2(delta) calcium channel subunit and its correlation with allodynia in spinal nerve-injured rats. J Neurosci. (2001) 21(6):1868–75. 10.1523/JNEUROSCI.21-06-01868.2001 11245671 PMC6762626

[159] van Deventer H Bernard S. Use of gabapentin to treat taxane-induced myalgias. J Clin Oncol. (1999) 17(1):434–5. 10.1200/JCO.1999.17.1 10458265

[160] Matsumoto M Inoue M Hald A Xie W Ueda H. Inhibition of paclitaxel-induced A-fiber hypersensitization by gabapentin. J Pharmacol Exp Ther. (2006) 318(2):735–40. 10.1124/jpet.106.103614 16687474

[161] Shinde SS Seisler D Soori G Atherton PJ Pachman DR Lafky J Can pregabalin prevent paclitaxel-associated neuropathy?—an ACCRU pilot trial. Support Care Cancer. (2015) 24:547–53. 10.1007/s00520-015-2807-5 26155765

[162] Gupta K Srikanth K Girdhar KK Chan V. Analgesic efficacy of ultrasound-guided paravertebral block versus serratus plane block for modified radical mastectomy: a randomised, controlled trial. Indian J Anaesth. (2017) 61(5):381–6. 10.4103/ija.IJA_62_17 28584346 PMC5444215

[163] Syal K Chandel A. Comparison of the post-operative analgesic effect of paravertebral block, pectoral nerve block and local infiltration in patients undergoing modified radical mastectomy: a randomised double-blind trial. Indian J Anaesth. (2017) 61(8):643–8. 10.4103/ija.IJA_81_17 28890559 PMC5579854

[164] van Helmond N Steegers MA Filippini-de Moor GP Vissers KC Wilder-Smith OH. Hyperalgesia and persistent pain after breast cancer surgery: a prospective randomized controlled trial with perioperative COX-2 inhibition. PLoS One. (2016) 11(12):e0166601. 10.1371/journal.pone.0166601 27935990 PMC5147830

[165] Ilfield BM Finneran J Swisher MW Said ET Gabriel RA Sztain JF Preoperative ultrasound-guided percutaneous cryoneurolysis for the treatment of pain after mastectomy: a randomized, participant- and observer-masked, sham-controlled study. Anesthesiology. (2022) 137:529–42. 10.1097/ALN.0000000000004334 35929983

[166] Tasmuth T Härtel B Kalso E. Venlafaxine in neuropathic pain following treatment of breast cancer. Eur J Pain. (2012) 6(1):17–24. 10.1053/eujp.2001.0266 11888224

[167] Abbas DN Reyad R. Thermal versus super voltage pulsed radiofrequency of stellate ganglion in post-mastectomy neuropathic pain syndrome: a prospective randomized trial. Pain Physician. (2018) 21(4):351–62. 10.36076/ppj.2018.4.351 30045592

[168] Marret E Vigneau A Salengro A. Effectiveness of analgesic techniques after breast surgery: a meta-analysis. Ann Fr Anesth Reanim. (2006) 25(9):947–54. 10.1016/j.annfar.2006.05.006 16926089

[169] Fahy A Jakub JW Dy BM Eldin NS Harmsen S Sviggum H Paravertebral blocks in patients undergoing mastectomy with or without immediate reconstruction provides improved pain control and decreased postoperative nausea and vomiting. Ann Surg Oncol. (2014) 21:3284–9. 10.1245/s10434-014-3923-z 25034821

[170] Glissmeyer C Johnson Q Sherman B Glissmeyer M Garreau J Johnson N. Effect of paravertebral nerve blocks on narcotic use after mastectomy with reconstruction. Am J Surg. (2015) 209(5):881–3. 10.1016/j.amjsurg.2015.01.013 25886701

[171] Rao R Jackson RS Rosen B Brenin D Cornett W Fayanju OM Pain control in breast surgery: survey of current practice and recommendations for optimizing management-American society of breast surgeons opioid/pain control workgroup. Ann Surg Oncol. (2020) 27(4):985–90. 10.1245/s10434-020-08197-z 31965373 PMC7241428

[172] Gong Y Tan Q Qin Q Wei C. Prevalence of postmastectomy pain syndrome and associated risk factors: a large single-institution cohort study. Medicine (Baltimore). (2020) 99(20):e198354. 10.1097/MD.0000000000019834 PMC7253604 32443289

[173] Kaur N Kumar A Saxena AK Grover RK. Pregabalin in the treatment of postmastectomy chronic pain: results of an open label, single-arm clinical study. Breast J. (2019) 25(3):465–8. 10.1111/tbj.13242 30916427

[174] Broyles J Tuffaha S Williams E Glickman L George TA Lee Dellon A. Pain after breast surgery: etiology, diagnosis, and definitive management. Microsurgery. (2016) 36(7):535–8. 10.1002/micr.30055 27043853

